# Evidence on the Heroin-Mediated Impairment of the Oxidative Status of Erythrocytes

**DOI:** 10.1155/2022/3996051

**Published:** 2022-09-28

**Authors:** Mohammad A. Bani-Ahmad, Ayman G. Mustafa, Abdelraheem A. Bani Ahmad, Islam E. Alkhazali, Ahmad A. Rahim

**Affiliations:** ^1^Department of Medical Laboratory Science, Faculty of Applied Medical Science, Jordan University of Science and Technology, Irbid, Jordan; ^2^Department of Basic Medical Sciences, College of Medicine, QU Health, Qatar University, Doha, Qatar

## Abstract

Away from hemorheological properties, the effect of heroin addiction on erythrocytes is poorly investigated. This study aimed to investigate the oxidative impacts of heroin administration on erythrocytes. Study subjects included chronic intravenous heroin addicts and control subjects. Hematological analysis and redox parameters were measured, including serum concentration of methemoglobin ([MethHb]), serum glutathione peroxidase-1 ([GPX-1]), serum glutathione peroxidase (GPX) activity, erythrocytic protein carbonyl content, and oxidized to reduced glutathione (GSSG/GSH) ratio. Hematological analysis revealed that addicts had a significantly higher red cell distribution width, consistent with the mild anisocytosis and poikilocytosis of erythrocytes. As compared to control subjects, significantly higher levels of serum [Met-Hb], [GPX-1], and GPX activity (*p* < 0.001) were reported among addicted subjects. A significant association between [MetHb] and GPX activity was observed with *r* = 0.764 (*p* < 0.001). Furthermore, significantly higher erythrocytic protein carbonyl contents and GSSG/GSH ratio were evident among heroin addicts (*p* < 0.005) that were significantly associated with *r* = 0.429 (*p*=0.01). Results demonstrate preliminary evidence that heroin addiction is implicated in impaired redox status of erythrocytes. Considering the pharmacokinetics of heroin, erythrocytic antioxidant mechanisms, and turnover rate, further investigation is required to evaluate the extent and clinical outcomes, especially upon over-dose administration.

## 1. Introduction

Opioids are analgesics that have been used in the management of painful disorders [1]. Despite these medications being used mainly for relieving acute pain conditions, their use is highly addictive in chronic diseases [2]. Physiological dependence and drug tolerance are significant obstacles to the continuous and long-term administration of opioids [3]. Heroin is the most abused opioid and has become a global problem [4]. Due to physiological tolerance and dependence, life-threatening complications may develop [2]. An overdose of administrated heroin is considered the primary cause of death among addicted subjects [5].

The destructive impact of heroin is mediated by oxidative damage to cellular constituents, including proteins, lipids, and nucleic acids [6]. Primarily, this results from an over-generation of reactive oxygen species that leads to impaired redox hemostasis [6, 7]. Furthermore, heroin administration is linked to the depletion of antioxidant compartments associated with the generation of reactive oxygen species [8, 9].

Heroin-mediated oxidative stress on cellular components of the blood is poorly investigated. Previously, it has been reported that chronic heroin addiction is associated with oxidative damage to platelets, as evident by increased protein carbonylation and lipid peroxidation in platelet lysates obtained from chronic heroin-addicted subjects [10]. This study aimed to investigate the impact of heroin administration on the redox status of erythrocytes. A complete blood count and a blood film examination were conducted to evaluate red cell indices and their morphological properties among chronic heroin-addicted subjects. Furthermore, serum and cellular markers of the erythrocytic redox status were investigated.

## 2. Materials and Methods

### 2.1. Study Subjects and Samples Collection

A total of thirty-five (*n* = 35) subjects with chronic addiction to intravenously administrated heroin were included in the study. Included subjects were addicts who were admitted to the addiction recovery center of the Drug Enforcement Administration/Public Security Department. Subjects were exclusively addicted to heroin with no abuse of other illicit drugs and no chronic diseases, including hypertension and diabetes mellitus. The subjects' medical profiles revealed that none were taking any medications, including antioxidants. For comparative purposes, age and sex-matched control subjects of nonaddicted subjects (*n* = 30) were included.

Under aseptic conditions, venous blood samples were withdrawn from participants: ethylenediaminetetraacetic acid (EDTA), plain blood samples (*n* = 20), and sodium heparinized blood (*n* = 15). EDTA blood samples were used for hematological analysis while plain blood samples were used to obtain serum. Red cell lysates were prepared from heparinized blood samples.

Before their inclusion, participants were informed about the study to obtain their consent. Following their approval, participants were asked to read and sign a consent form. This study was reviewed and approved by an institutional review board (IRB) at Jordan University of Science and Technology.

### 2.2. Determination of Red Cells Indices

Red cell indices were determined by a complete blood count (CBC) analysis that was conducted using an automated cell counter (Beckman Coulter LH 780 Analyzer). Measured red cell indices included red cell count (RBC's), hemoglobin concentration ([Hb]), hematocrit (Hct), mean corpuscular hemoglobin (MCH), mean corpuscular volume (MCV), mean corpuscular hemoglobin concentration (MCHC), and red cell distribution width (RDW). Furthermore, morphological evaluation of erythrocytes was conducted by microscopic examination of Giemsa-stained blood films, using a compound microscope (Nikon, Japan).

### 2.3. Serum Methemoglobin (MetHb) and Glutathione Peroxidase-1 (GPX-1) Concentration

Serum MetHb and GPX-1 concentration were, quantitatively, measured by enzyme-linked immunosorbent assay (ELISA) using commercially available kits (Cusabio, Texas, USA). The experimental work was conducted in accordance with the manufacturer's instructions. Briefly, serum was diluted to 1 : 200 and 1 : 1000 for the measurement of MetHb and GPX-1, respectively. A 100 *µ*L of each diluted serum samples were loaded and incubated for two hours at 37°C. After removing supernatants, 100 *µ*L of biotinylated antibodies were added and incubated at 37°C for one hour. After that, streptavidin-conjugated horseradish peroxidase was added, and the plate was incubated at 37°C for an hour. Finally, a stop solution was added, and optical density (OD) was measured at a wavelength (*λ*) of 450 nm. The ratio of serum methemoglobin concentration to total hemoglobin concentration (MetHb/Hb) was determined by dividing serum methemoglobin concentration by the total hemoglobin concentration (Hb).

### 2.4. Serum Glutathione Peroxidase (GPX) Activity

The enzymatic activity of GPX was measured using a commercially available kit (Abcam, Cambridge, UK). The assay is based on the colorimetric detection of NADPH absorbance at a wavelength of 340 nm. NADPH oxidization into NADP+ is associated with the reduction of oxidized glutathione (GSSG) into GSH by GPX activity. Measurement was conducted in accordance with manufacturer instructions. Results are expressed in mU/mL that defines the oxidization of 1.0 *μ*mol of NADPH to NADP^+^ per minute at 25°C.

### 2.5. Red Cell Lysate Preparation

Red cell lysates were prepared using commercially available lysis buffer (Abcam, Cambridge, UK). According to the manufacturer's instructions, 20 volume units of 1X lysis buffer were mixed with 1 volume unit of whole blood and incubated for 10 minutes at room temperature. The mixtures were then centrifuged at 400x for 5 minutes and the sediments were resuspended in phosphate buffer saline (PBS).

### 2.6. Protein Carbonyl Content Determination

Initially, protein concentration in red cell lysates was determined by the bicinchoninic acid (BCA) protein assay (Abcam, Cambridge, UK). In brief, 50 mL of red cell lysates were treated with 100 mL of working solution and incubated at 37°C for 60 minutes. Protein concentration was determined by a spectrophotometer at a wavelength (*λ*) of 562 nm.

Protein carbonyl contents were measured using a commercially available kit (Abcam, Cambridge, UK). According to the manufacturer's instructions, 100 microliters of standardized cell lysates (final protein concentration was 1 mg/dL) were incubated with an equal volume of dinitrophenyl hydrazine for 10 min at room temperature. After that, cell lysates were incubated with 30 mL of trichloroacetic acid (TCA) on ice for 5 minutes. Precipitates were double washed with 500 milliliters of cold acetone and then allowed to dissolve in guanidine hydrochloride. Final solutions were loaded into a 96-well plate and the optical density was determined at a wavelength (*λ*) of 375 nm.

### 2.7. Oxidized to Reduced Glutathione (GSSG/GSH) Ratio

The ratio of GSSG/GSH was determined using a commercially available kit (Abcam, Cambridge, UK). Initially, cell lysates were deproteinized by the TCA kit (Abcam, Cambridge, UK). The GSH concentration was determined by incubating 50 *µ*l of deproteinized cell lysates with 50 *µ*L of thiol green solution for 10 minutes at room temperature. In a second set, total GSH was determined by mixing thiol green solution with the GSSG probe in 1 : 25 dilution. 50 *µ*l of the mixture were incubated with 50 *µ*l of deproteinized cell lysates for 10 minutes at room temperature. The final concentration was determined at an excitation/emission wavelength of 490/520 nm. The GSSG concentration was calculated by subtracting GSH concentration from total GSH, and then the GSSG/GSH ratio was mathematically calculated.

### 2.8. Statistical Analysis

Data were analyzed using the statistical package for the social sciences (SPSS) version 22. The independent students *t*-test was used for comparative purposes. A correlation analysis was conducted using parametric Pearson's (*r*) correlation analysis and nonparametric Spearman's (rho) coefficient to test the linearity and tonicity of relationships, respectively. Statistical significance was considered whenever the *p* value was less than 0.05. Graphs were prepared using GraphPad Prism 8 software.

## 3. Results

A total of seventy (*N* = 70) male subjects participated in the study, of whom thirty-five subjects were addicted to intravenous (IV) heroin administration. All subjects were chronically addicted subjects with an average total duration of addiction (TDA) of 9.3 ± 1.8 years, an average administered dose of 0.80 ± 0.22 mg, and an average time of the latest administrated dose (TLAD) of 8.7 ± 1.5 days before their enrolment and blood sampling.

Except for RDW, the hematological analysis revealed that all red cell indices of all study subjects were within the standard reference intervals with no significant differences between addicted and control subjects (Table 1). The mean of RDW among heroin-addicted was 14.3 ± 0.1%, which is slightly, but significantly, higher than its corresponding mean among control subjects (13.4 ± 0.3%) (*p*=0.005). Blood film examination defined a mild presence of a slightly macrocytic population of erythrocytes and a mild presence of lemon-shaped cells and helmet cells.

**Figure 1:**
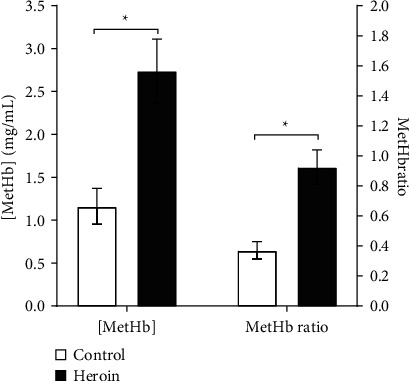
Comparative analysis of serum (a) methemoglobin concentration ([Met-Hb]) and (b) methemoglobin ratio to total hemoglobin between chronic heroin-addicted subjects and control subjects. Results are presented as mean ± SEM in milligram per milliliter (mg/mL). ^*∗*^indicates a significant comparative analysis with *p* < 0.001.

Regarding the serum concentration of Met-Hb, the average concentration among addicted subjects was 2.74 ± 0.37 mg/mL. This is significantly higher than the corresponding average concentration among control subjects, at 1.16 ± 0.21 mg/mL (*p*=0.000). Furthermore, the MetHb/Hb among addicted subjects was 1.62 ± 0.20% which is significantly higher (*p* < 0.001) than the corresponding ratio among control subjects (0.65 ± 0.10%).  Figure 1 demonstrates the comparative analysis results of serum Met-Hb concentration and MetHb/Hb ratio.

**Figure 2:**
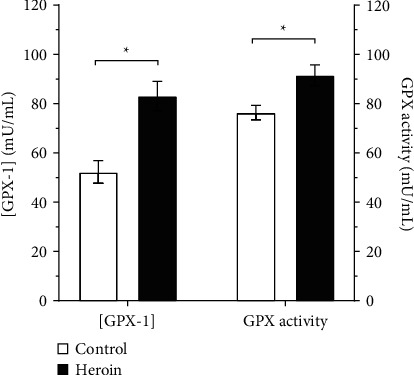
Comparative analysis of serum (a) glutathione peroxidase-1 (GPX-1) concentration and (b) glutathione peroxidase (GPX) activity between chronic heroin-addicted subjects and control subjects. Results are presented as mean ± SEM in milliunits per milliliter (mU/mL). ^*∗*^indicates a significant comparative analysis with *p*=0.001.

**Figure 3:**
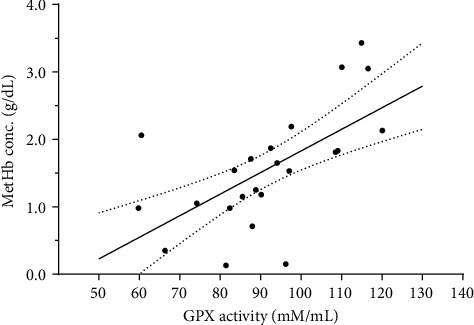
Correlation analysis of serum GPX activity and serum MetHb to total hemoglobin ratio among heroin-addicted subjects. A significant direct and linear association was evident with *r* = 0.777, *p*=0.001. The continuous line defines the best fit linear correlation model while the dotted lines define the standard error means (SEM).

Similarly, addicted subjects were having significantly higher GPX-1 concentration (83.1 ± 5.9 mU/mL) and total GPX activity (91.5 ± 4.2 mU/mL) as compared to their corresponding values among control subjects (*p*=0.001 and *p*=0.006, respectively). Among control subjects, the GPX-1 concentration was 52.2 ± 4.6 mU/mL and the GPX activity was 76.4 ± 2.9 mU/mL. Results of serum GPX-1 and GPX activities are illustrated in Figure 2. As demonstrated in Figure 3, among addicted subjects, there was a significant linear correlation of GPX activity with MetHb/Hb ratio (*r* = 0.764, *p*=0.001). On the other hand, no significant correlation was observed between these parameters among control subjects (*p* > 0.0.5).

**Figure 4:**
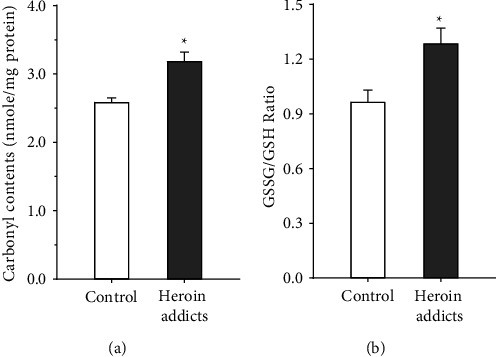
Comparative analysis of (a) protein carbonyl contents (nmol/mg protein) and (b) GSSG/GSH ratio in red cell lysates from study subjects: chronic heroin-addicted subjects and control subjects. Results are presented as mean ± SEM. ^*∗*^indicates a significant comparative analysis with *p*=0.001.

**Figure 5:**
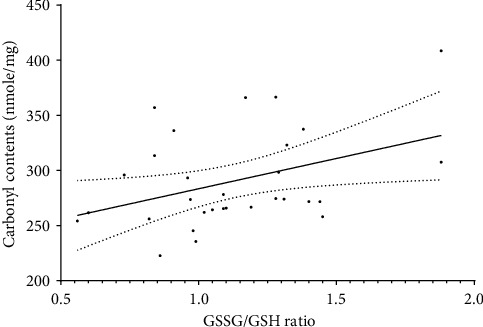
Correlation analysis of protein carbonyl contents and GSSG/GSH ratio in red cell lysates from study subjects. A significant, linear, and direct relationship was demonstrated with *r* = 0.429, *p*=0.01. The solid line defines the best fit line for the correlation model while the dotted lines define the standard error means (SEM).

Analysis of erythrocyte lysates revealed that heroin-addicted subjects had significantly higher average protein carbonyl contents (311.9 ± 12.4 nmol/mg) as compared to 268.8 ± 5.0 nmol/mg among control subjects (*p*=0.004). Similarly, addicted subjects had a significantly higher average GSSG/GSH ratio (1.28 ± 0.08%) in comparison to an average ratio of 0.97 ± 0.06% among control subjects (*p*=0.005). Results are illustrated in Figure 4. Pearson's (*r*) correlation analysis demonstrated a significant direct association between protein carbonyl content and GSSG/GSH ratio with an *r* = 0.429 (*p*=0.01). The correlation analysis is demonstrated in Figure 5.

To investigate the contribution of addiction profiles (DA, TLAD, and AD) to the study redox parameters, Pearson's correlation analysis was conducted (Table 2). Results revealed significant inverse linear associations of the DA with both serum GPX-1 concentration (*r* = −0.594, *p*=0.02) and GSSG/GSH ratio (*r* = −0.612, *p*=0.01).

## 4. Discussion

Heroin administration is associated with an impaired redox status, that is, primarily, mediated by the generation of reactive oxidizing species with harmful impacts on cellular compartments, including proteins, lipids, and nucleic acids [11]. Several studies have demonstrated the modulation of rheological features of blood and its components in response to heroin administration [12]. A major emphasis was on the immunological properties of leukocytes [13]. Chronic heroin addiction is associated with oxidative stress on platelet lipids and proteins [10]. Herein, we aimed to investigate the effects of oxidative stress on the most prominent cellular blood component, erythrocytes. The modulatory impacts of heroin addiction on the redox status of red cells are poorly studied. Herein, we aimed to investigate the possible impairment of erythrocytic redox status in response to chronic heroin administration.

An initial comparative analysis of red cell indices was conducted to evaluate the functional and morphological features of erythrocytes among study subjects. The insignificant differences in most red cell parameters among chronic heroin addiction subjects may represent no or low harmful effects of heroin administration on red cell viability and integrity. However, the significant elevation of RDW and the morphological abnormalities among heroin-addicted subjects may be attributed to the pathological consequences of heroin administration on erythrocytes. These findings agree with the previously reported macrocytosis and increased RDW values among heroin abusers [14]. The diagnostic and prognostic value of RDW in the context of a variety of pathological situations has been defined [15]. Increased RDW can result from an increased turnover of large erythroid cells and/or an increased conscience of circulatory erythrocytes [15]. In turn, the high turnover rate of erythrocytes is a consequence of a reduced red cell mass and decreased perfusion in pathological contexts, including hemolytic crises [16]. Accordingly, the reported morphological abnormalities, among addicted subjects, may explain the increase in RDW in response to the harmful impacts of heroin administration on erythrocytes' viability and integrity.

Erythrocytes are continuously exposed to various endogenous and exogenous sources of oxidizing agents that influence the generation of ROS [17, 18]. Furthermore, in pathological situations, immune cells produce and release ROS that can be taken up by erythrocytes [17]. Favoring to the possession of antioxidant mechanisms, erythrocytes are highly capable of neutralizing the harmful oxidizing effects of ROS [17, 18]. However, if the capacity of antioxidant systems is exceeded, oxidative damage to cellular compartments occurs [19]. Proteins are the most common targets for oxidative-mediated denaturation in response to intracellular and extracellular ROS, where the extent of damage is proportional to the concentration of the targeted component [20, 21]. Since it is the most prominent cytoplasmic compartment of erythrocytes, hemoglobin is a primary target of oxidative ROS in erythrocytes [18].

Upon exposure to oxidative stress, hemoglobin is converted into methemoglobin, leading to Heinz bodies (oxidized methemoglobin inclusion) formation. Heinz bodies make erythrocytes vulnerable to immune-mediated intravascular hemolysis. Intravascular hemolysis is associated with the release of intracellular contents, including methemoglobin, into the circulatory. Therefore, methemoglobin level has been suggested as a prognostic marker to follow up on severe immune-mediated hemolytic anemia [22]. The significantly higher serum methemoglobin among chronic heroin-addicted subjects may suggest that their erythrocytes were exposed to overwhelming oxidative stress, which exceeded their antioxidant capacity.

Serum GPX-1 concentration findings define the potential oxidative stress on erythrocytes in response to heroin addiction. GPX-1 is the most abundant member of the GPX family that is mainly implicated in the host's protection against oxidative stress [23, 24]. GPX-1 is an intermediate in the protection of hemoglobin against oxidative damage [23]. GPX-1 mediates the detoxification of H_2_O_2 by_ utilizing glutathione (GSH) as a reducing agent that, in turn, is converted into its oxidized form (GSSG) [25]. The higher serum GPX-1 levels among heroin-addicted subjects correlate significantly with serum methemoglobin concentrations. It is worth mentioning here that the upregulation of plasma GPX levels is an adaptive protective response that protects host tissues against oxidative damage [26]. In addition to the influence of [GPX-1], the significantly higher serum GPX activity is another supportive piece of evidence that defines the harmful oxidative impacts of heroin administration. Circulatory GPX is either a cellular cGPX/GPX-1 (erythrocytic) or extracellular eGPX/GPX-3 (in plasma). In addition to other potential sources, GPX-3 is mainly released by the kidney [27].

Based on the obtained findings on serum oxidative parameters, we primarily concluded that erythrocytes are possible targets of harmful oxidative damage by heroin. To further support that, erythrocytic oxidative measures were investigated. The significantly higher erythrocytic protein carbonyl content and GSSG/GSH ratio among heroin-addicted subjects were definitive of the oxidative impacts of heroin administration on erythrocytic constituents that include, primarily, proteins. Furthermore, the significant linear association between protein carbonyl content and GSSG/GSH ratio is supportive of the oxidative stress implicated in erythrocytes. ROS in erythrocytes can be either a result of either an endogenous formation or uptaken by erythrocytes following their generation by immune cells. These ROS, including superoxide free radicals (H_2_O_2_), are highly capable of inducing the Fenton–Haber–Weiss reaction, where toxic hydroxyl radicals (OH) can be generated by reacting with transition metallic ions, including iron [28]. It is worth mentioning that erythrocytes encompass most of the body's iron content [29].

The lack of significant association between most redox parameters and the addiction profile of study subjects can be attributed to several factors. Firstly, the enigmatic administrated dose that is a result of the variable purity of “illicit street heroin” [30]. Variable heroin purity has been identified as the possible cause of overdose-associated fatalities among heroin-addicted subjects [5]. Secondly, the molecular heterogeneity of the various enzymatic and nonenzymatic antioxidant mechanisms possessed by erythrocytes [18]. The significant inverse association of [GPX-1] and DA agrees with the previously reported correlation between the duration of chronic heroin addiction and the gradual decrease of plasma levels of antioxidants [31]. Notably, potent heroin metabolites are characterized by significantly prolonged plasma bioavailability in response to enterohepatic cycling, resulting in long-term oxidative-mediated toxicity [32].

Herein, we provide preliminary evidence that heroin addiction is associated with impaired redox status in erythrocytes. The dose and purity of administrated heroin among our study subjects, the antioxidant mechanisms possessed by erythrocytes, and their high turn-over rate may contribute to the few harmful consequences on erythrocytes. However, considering the pharmacokinetics of heroin, high doses of heroin can be associated with severe impacts on erythrocytic redox status and, subsequently, cellular integrity and viability. Earlier studies have reported significantly higher concentrations of 6-acetylmorphine, free morphine, and total opiates in the blood among overdose subjects [33]. Accordingly, further investigation is to evaluate the extent and dose-dependence of heroin-associated toxicity to erythrocytes. Our finding may aid in a better understanding of the pathologic consequences of overdose administration and define an intermediate in the serious health-threatening and mortality among overdose subjects.

## Figures and Tables

**Table 1:** Comparison of red cell indices among chronic heroin addicts and control subjects: there was no significant difference between control and heroin-addicted subjects, in all red cell indices, except the RDW (*p* < 0.05).

Indices (unit)	Control (mean ± SEM)	Heroin (mean ± SEM)	*p* value
RBC count (10^6^ cell/*μ*L)	5.3 ± 0.1	5.2 ± 0.2	0.864
Hb (g/dL)	15.6 ± 0.3	15.7 ± 0.2	0.834
Hct (%)	45.6 ± 0.5	46.1 ± 0.7	0.595
MCV (fL)	85.9 ± 1.1	87.4 ± 1.4	0.410
MCH (pg)	29.4 ± 0.4	29.8 ± 0.6	0.594
MCHC (g/dL)	34.2 ± 0.2	34.0 ± 0.2	0.462
RDW (%)	13.4 ± 0.3	14.3 ± 0.1	0.005^*∗*^

Hb, hemoglobin concentration; Hct, hematocrit; MCV, mean corpuscular volume; MCH, mean corpuscular hemoglobin; MCHC, mean corpuscular hemoglobin concentration; RDW, red cell distribution width. Results are presented as mean ± SEM. ^*∗*^It is statistically significant with *p* < 0.05

**Table 2:** Correlation analysis of studied serum redox markers and addiction profile data of chronic heroin addiction subjects. A significant correlation was evident for [GPX-1] and carbonyl contents with the duration of addiction (*p* < 0.05).

Parameters	Correlation	DA	TLAD	AD
[Met-Hb]	Coefficient (r)	−0.260	0.315	-0.206
	*p* value	0.27	0.14	0.38

[GPX-1]	Coefficient (r)	−0.509	0.147	0.029
	*p* value	0.02^*∗*^	0.54	0.90

GPX activity	Coefficient (r)	−0.290	0.282	−0.033
	*p* value	0.22	0.23	0.90

Carbonylcontents	Coefficient (r)	−0.612	−0.075	−0.040
	*p* value	0.01^*∗*^	0.80	0.90

GSSG/GSH	Coefficient (r)	0.431	−0.135	0.131
	*p* value	0.11	0.63	0.65

DA: duration of addiction in years; TLAD: timing of the lad administrated dose (days); AD: administrated dose. ^*∗*^It is statistically significant with *p* < 0.05

## Data Availability

The datasets used to support the findings of this study are available from the corresponding author upon request.
